# Eugenol and carvacrol attenuate brain d-galactose-induced aging-related oxidative alterations in rats

**DOI:** 10.1007/s11356-022-18984-8

**Published:** 2022-02-19

**Authors:** Ali H. El-Far, Hadeer H. Mohamed, Doaa A. Elsabagh, Shymaa A. Mohamed, Ahmed E. Noreldin, Soad K. Al Jaouni, Abdelwahab A. Alsenosy

**Affiliations:** 1 Department of Biochemistry, Faculty of Veterinary Medicine, Damanhour University, Damanhour, 22511 Egypt; 2 Molecular Biology, Molecular biology unit, Medical Technology Center, Medical Research Institute, Alexandria University, Alexandria, Egypt; 3 Histology and Cytology Department, Faculty of Veterinary Medicine, Damanhour University, Damanhour, 22511 Egypt; 4 Department of Hematology/Pediatric Oncology, Yousef Abdulatif Jameel Scientific Chair of Prophetic Medicine Application, Faculty of Medicine, King Abdulaziz University, Jeddah, 21589 Saudi Arabia

**Keywords:** Aging, Apoptosis, Anti-aging, Eugenol, Carvacrol

## Abstract

Aging represents the accumulation of progressive changes in a human being over time and can cover physical, psychological, and social changes. It is an oxidative stress-associated process that progresses with age. The antioxidant activity of either eugenol (EU) or carvacrol (CAR) for aging in rats induced by d-gal for 42 days was investigated in the current study using 10 and 20 mg of EU/kg/day/orally, while CAR was supplemented by 40 and 80 mg /kg/day/orally. Biochemical, mRNA expression, and histopathological assessments of brain samples evaluated the oxidative alterations induced by d-gal and the protective role of EU and CAR. Results showed that d-gal was causing oxidative alternation of the brain that was recognized via upregulation of *p53* and *p21* mRNA expression levels, as aging markers and *Bax* mRNA expression level, as an apoptotic marker. Also, the results observed alterations in the levels of biochemical markers as creatine phosphokinase (CPK) and triacylglycerol (TAG), besides, enhancement of brain antioxidant capacity. Finally, these results compared with the groups treated with EU and CAR to observe that the EU and CAR potentially attenuate these aging-related oxidative alterations in a dose-dependent manner. Finally, we can conclude that EU and CAR supplementations are considered promising natural protective compounds that could delay aging and maintain health.

## Introduction

Aging is a progressive physiological change due to oxidative stress leading to a reduction in the functional potential over the entire adult life span (Davalli et al. 2016). An imbalance between oxidants and antioxidants causes oxidative stress, reducing antioxidant ability and accumulation of reactive oxygen species (ROS), leading to oxidative cellular alterations in lipid, protein, and nucleic acids (Neki 2015). Exposure to toxins, the sun, harmful foods, pollution, and smoke can damage tissue due to ROS generation (Krafts 2010).

In nature, the main form of galactose is d-galactose (d-gal). Milk and dairy products are the primary natural source of galactose (Acosta and Gross 1995). Some fruits and vegetables like tomatoes, brussels sprouts, bananas, and apples also have free galactose (Gross and Acosta 1991). In addition, the lactose hydrolysate syrup, as a sweetener, has been intensively used in biscuits, confectionery, and some dairy desserts containing high galactose content (Williams 2003).

The d-gal-induced aging model has been widely used to study aging (Parameshwaran et al. 2010; El-Far et al. 2020b). Higher d-gal caused ROS and decreased brain antioxidant activity, brain senescence, and shortened the duration of life (Coelho et al. 2015). Also, during its metabolism, d-gal produces ROS and produces advanced glycation end-products (AGE) glycation products that eventually speed up the aging process (Bucala and Cerami 1992; Song et al. 1999).

Natural products have played a critical role in drug discovery, especially cancer and infectious diseases (El-Far 2015; El-Far et al. 2018, 2020a, b, 2021; Ashrafizadeh et al. 2020, 2021; Atanasov et al. 2021; Abadi et al. 2021; Mohsen et al. 2022). Eugenol (4-Allyl-2-methoxyphenol, EU) is a major phenolic component of clove oil (*Eugenia caryophyllata*) which has powerful antioxidant and radical-scavenging activities (Gülçin 2011). Another natural product is carvacrol (CAR), the phenolic monoterpenoid found in essential oils of oregano (*Origanum vulgare*), thyme (*Thymus vulgaris*), pepperwort (*Lepidium flavum*), wild bergamot (*Citrus aurantium bergamia*), and other plants (Sharifi-Rad et al. 2018). The high antioxidant activity of CAR is due to the hydroxyl group (OH), linked to the aromatic ring (Mondal et al. 2021). The present experiment aims to assist the ani-aging protective effect of EU and CAR in experimentally induced aging in rats’ brains by d-gal.

## Materials and methods

### Ethics statement

The study was approved in response to “NIH Guide for the Care and Use of Laboratory Animals” by the Faculty of Veterinary Medicine Ethics Committee of Damanhour University, Egypt.

### Experimental design

Fifty-six male Wistar rats between 90 and 110 g were housed in a standard laboratory conditions with a 12-h light/dark cycle and allowed to freely access food pellets (Table 1) and water. Rats were allocated into seven groups (*n*= 8 per group). In the control group, rats were subcutaneously injected with physiological saline solution (0.9%) daily. In comparison, the vehicle group was injected subcutaneously with physiological saline solution (0.9%) daily and orally supplemented with olive oil daily. Rats in the d-gal group were injected subcutaneously with 200 mg d-gal/kg body weight (B.W.) (Fan et al. 2017) dissolved in saline solution daily along with oral supplementation with olive oil. In the d-gal+EU10 group, rats were injected subcutaneously with 200 mg d-gal/kg B.W. dissolved in saline solution daily and orally supplemented with EU by a dose of 10 mg/kg B.W. (Mateen et al. 2019) dissolved in olive oil (Yogalakshmi et al. 2010), while in d-gal+EU20 group, rats were injected subcutaneously with 200 mg d-gal/kg B.W. daily dissolved in saline solution in addition to oral supplementation with EU by a dose of 20 mg/kg B.W. (Mateen et al. 2019) dissolved in olive oil (Yogalakshmi et al. 2010). Rats in the d-gal+CAR40 group were injected subcutaneously with d-gal (200 mg/kg B.W.) dissolved in saline solution daily plus oral supplementation with CAR by a dose of 40 mg/kg B.W. daily (Aristatile et al. 2009) dissolved in olive oil (Stojanović et al. 2019); on the other hand, in the d-gal+CAR80 group, rats were injected subcutaneously with d-gal (200/kg B.W.) daily dissolved in saline solution and orally supplemented with CAR by a dose of 80 mg/kg B. W daily (Aristatile et al. 2009) dissolved in olive oil (Stojanović et al. 2019). The experiment was spent for 42 days, and rats were weighed on day 42.

**Table 1:** Ingredients of the basal diet

Ingredients	g/kg diet
Corn flour	529.5
Casein	200
Sucrose	100
Soybean oil	70
Cellulose	50
Mineral mix	35
Vitamin mix	10
l-cystine	3
Choline	2.5

### Sampling

On day 42, the animals were anesthetized by isoflurane inhalation and euthanized by cervical dislocation. Blood samples were collected from rats’ veins. After centrifugation at 1000 ×g for 15 min at room temperature, clear sera were separated, labeled, and subjected to biochemical analyses.

Brain samples of the cerebellum and hippocampus were collected after surgical removal then flushed with phosphate buffer saline (PBS) to remove excess blood for histopathological, antioxidant parameters and mRNA expression assessment. Part of the brain samples was fixed in 4% paraformaldehyde dissolved in PBS for 48 h for sample fixation. Other parts were labeled and kept in −80°C for antioxidant status and mRNA expression assessments.

### Biochemical assessment

Serum samples were subjected to determination of total cholesterol (T.cholesterol), triacylglycerol (TAG), alanine aminotransferase (ALT, EC 2.6.1.2), aspartate aminotransferase (AST, EC 2.6.1.1), creatinine, creatine phosphokinase (CPK, EC 2.7.3.2), and lactate dehydrogenase (LDH, EC 1.1.1.27). Biochemical tests measured using Roche/Hitachi Cobas c 311, Cobas c 501/502 analyzers measured using the full automated system.

### Oxidative stress and antioxidant status assessment

Oxidative stress and antioxidant biomarkers were analyzed in brain homogenate 20% (w/v) using cooled 0.1 M phosphate buffer saline and subjected to determination of malondialdehyde (MDA) and total antioxidant capacity (TAC) levels and the activities of glutathione peroxidase (GPx; EC 1.11.1.9) and glutathione S-transferase (GST; EC 2.5.1.18) using commercial Biodiagnostic Co. (Giza, Egypt) kits. Protein concentrations of brain homogenates were assessed with the Bradford assay (5000002, Bio-Rad Laboratories, Watford, UK) to standardize biochemical parameters (Bradford 1976).

### Gene expression assessment by real-time polymerase chain reaction (RT-PCR)

According to the manufacturer’s kit, total RNA was extracted from the tissue samples (Easy spin TM kit Total RNA Extraction Kit, INTRON Biotechnology, Korea). The purities and concentration of RNA were measured by A Nanodrop spectrophotometer (Genway Nanodrop, Germany). 1 μg of RNA (260/280 ratio = 1.8 – 2.0) used for the transcription of cDNA using RT-Premix Kit (INTRON, Biotechnology, Korea.). 2 μl of RT product were mixed with 10 μl of SYBR-Green master mix (INTRON, Biotechnology, Korea) and 0.5 mM of each forward and reverse primer (Table 2) and nuclease-free water in a final volume of 20 μl. All reactions were performed on a 7500 Applied Biosystems, USA, with the following conditions: 95 °C for 10 min, followed by 40 cycles at 95 °C for 15 s, 58 °C for 15 °C, and 72 °C for 30 s. Relative expression of mRNA was normalized to β-actin as housekeeper gene. The fold changes of mRNA expression were calculated with the 2^-ΔΔCt^ method described by Livak and Schmittgen (2001).

**Table 2:** Primer’s sequence

Genes	Primers 5→ 3	Accession number	Tm	Product size
*p53*	F: CCCACCATGAGCGTTGCT	NM_030989.3	60.36	116
	R: CCACCCGGATAAGATGTTGG		62.46	
*p21*^*CIP1/WAF1*^	F: GACCTGTTCCACACAGGAGCAAAG	NM_080782.3	63.82	145
	R: GTCTCAGTGGCGAAGTCAAAGTTC		62.07	
*Bax*	F: GCGAATTGGCGATGAACTG	NM_017059.2	57.78	216
	R: ATGGTTCTGATCAGCTCGG		56.92	
*β-actin*	F: GCCGTCTTCCCCTCCATCGTG	NM_031144.3	65.10	358
	R: TACGACCAGAGGCATACAGGGACAAC		65.85	

### Histopathological assessment

After flushing with PBS (pH 7.4) and fixing in 4 % paraformaldehyde dissolved in PBS for 48 h, the fixed specimens were processed by the conventional paraffin embedding technique, which included the dehydration through ascending grades of ethanol, clearing in three changes of xylene and melted paraffin and finally embedding in paraffin wax at 65°C. Four-micrometer thick sections were stained by hematoxylin and eosin (Bancroft and Layton 2013). Micrographs of the sections were taken with a digital camera (Leica EC3, Leica, Germany) connected to a microscope (Leica DM500).

### Statistical analysis

A one-way ANOVA with Tukey’s post hoc multiple range tests was used for the data analysis using a GraphPad Prism v.5 (https://www.graphpad.com/), accessed on 10 March 2021 (GraphPad, San Diego, CA, USA). All declarations of significance depended on *P* < 0.05.

## Results

### Body weight

**Fig. 1:**
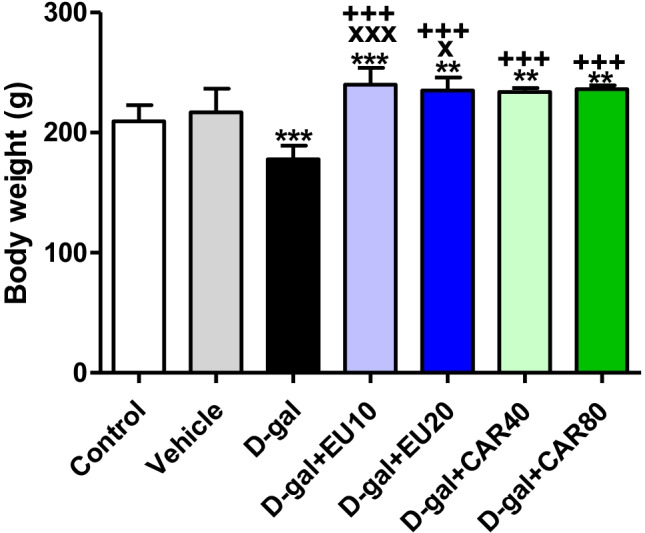
Body weight. Data were analyzed with a one-way ANOVA followed by Tukey’s multiple comparison test. ^**^P < 0.01 and ^***^P < 0.001 vs the control. ^x^*P* < 0.05 and ^xxx^*P* < 0.001 vs vehicle. ^+++^*P* < 0.001 vs d-gal. Error bars represent mean ± SD. *n*= 8.

The body weight in the d-gal group was significantly decreased (*P* < 0.001) compared with the control group. On the other hand, in the d-gal+EU10 (*P* < 0.001), d-gal+EU20 (*P* < 0.01), d-gal+CAR40 (*P* < 0.01), and d-gal+CAR80 (*P* < 0.01) groups, the body weight exhibited a significant increase in comparison with the control group (Fig. 1).

In comparison with the vehicle group, the body weight in the d-gal+EU10 (*P* < 0.001) and the d-gal+EU20 (*P* < 0.05) groups was markedly increased. Also, the body weight in the d-gal+EU10, d-gal+EU20, d-gal+CAR40, and d-gal+CAR80 groups exhibited significant increases (*P* < 0.001) in comparison with the d-gal group.

### Biochemical assessment

**Fig. 2:**
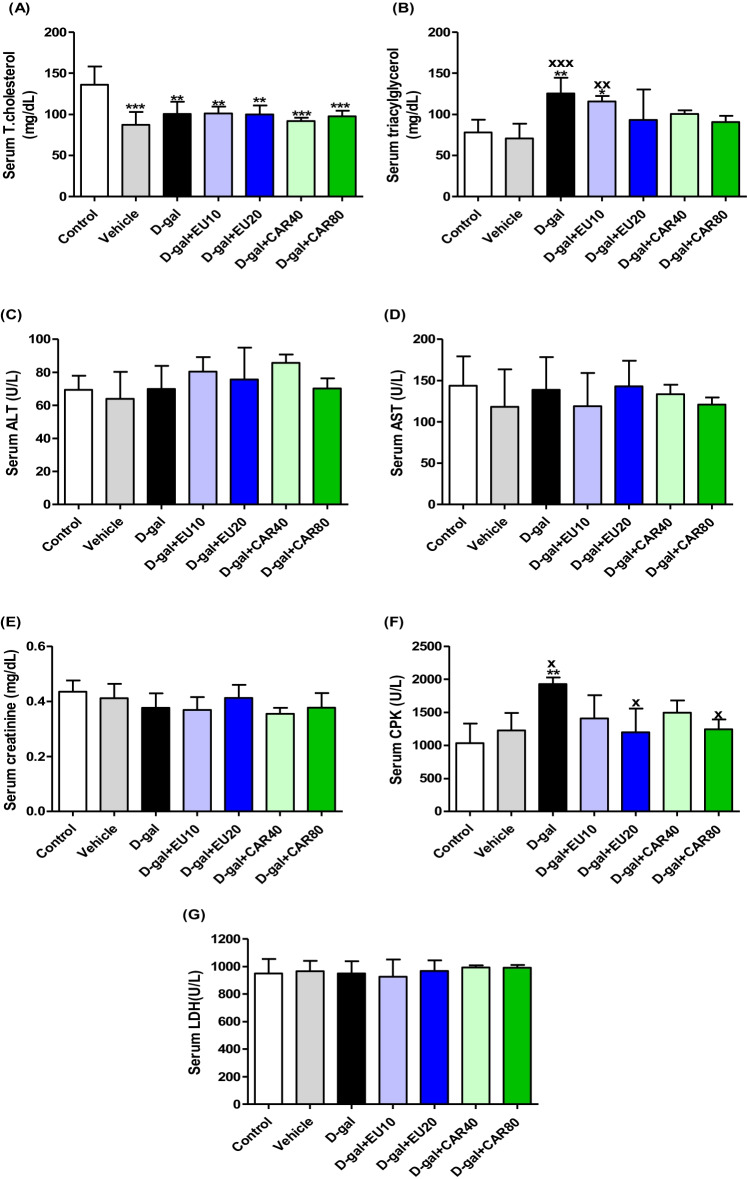
Biochemical assessment of serum **A** total cholesterol (T.cholesterol), **B** triacylglycerol, **C** alanine aminotransferase (ALT), **D** alanine aminotransferase (AST), **E** creatinine, **F** creatine phosphokinase (CPK), and **G** lactate dehydrogenase (LDH). Data were analyzed with a one-way ANOVA followed by Tukey’s multiple comparison test. ^*^P < 0.05, ^**^P < 0.01, and ^***^P < 0.001 vs the control. ^x^*P* < 0.05, ^xx^*P* < 0.01, and ^xxx^*P* < 0.001 vs vehicle. Error bars represent mean ± SD. *n*= 7.

The serum total cholesterol levels in the d-gal (*P* < 0.01), d-gal+EU10 (*P* < 0.01), d-gal+EU20 (*P* < 0.01), vehicle (*P* < 0.001), d-gal+CAR40 (*P* < 0.001), and d-gal+CAR80 (*P* < 0.001) groups were significantly decreased compared with the control group (Fig. 2A).

The serum TAG levels in the d-gal group were significantly increased (*P* < 0.01) compared with the control group. Also, in the d-gal+EU10, its level exhibited a significant increase (*P* < 0.05) in comparison with the same group. TAG levels were markedly raised increased in d-gal (*P* < 0.001) and d-gal+EU10 (*P* < 0.01) groups in comparison with the vehicle group (Fig. 2B). Serum CPK activities in the d-gal group were significantly increased compared with the control (*P* < 0.01) and vehicle (*P* < 0.05) groups. In the d-gal+EU20 and d-gal+CAR80, its levels were significantly decreased (*P* < 0.05) in comparison with the vehicle group.

### Oxidative stress and antioxidant status

**Fig. 3:**
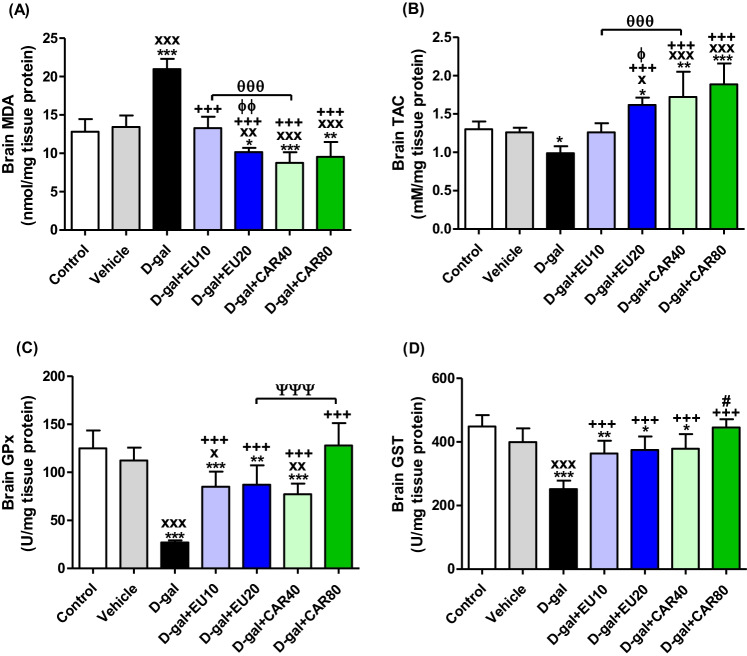
Oxidative stress and antioxidant status in brain. **A** Malondialdehyde (MDA), **B** Total antioxidant capacity (TAC), **C** Glutathione peroxidase (GPx), and **D** Glutathione S-transferase (GST). Data were analyzed with a one-way ANOVA followed by Tukey’s multiple comparison test. ^*^P < 0.05, ^**^P < 0.01, and ^***^P < 0.001 vs the control. ^x^*P* < 0.05, ^xx^*P* < 0.01, and ^xxx^*P* < 0.001 vs vehicle. ^+++^*P* < 0.001 vs d-gal. ^ɸ^*P* < 0.05 and ^ɸɸ^*P* < 0.01 vs d-gal+EU10. ^#^*P* < 0.05 vs d-gal+CAR40. ^θθθ^*P* < 0.001 for d-gal+EU10 and d-gal+CAR40 comparison. ^ΨΨΨ^*P* < 0.001 for d-gal+EU20 and d-gal+CAR80 comparison. Error bars represent mean ± SD. *n*= 7.

Induction of aging by d-gal significantly (*P* < 0.001) increased MDA (Fig. 3A), the product of oxidative stress, compared with control and vehicle groups. MDA levels in brain homogenates were significantly (*P* < 0.001) decreased in d-gal+EU10, d-gal+EU20, d-gal+CAR40, and d-gal+CAR80 groups compared with the d-gal group. TAC levels (Fig. 3B) GPx (Fig. 3C) and GST (Fig. 3D) activities were significantly increased in the same groups compared with the d-gal group to neutralize the oxidative stress process.

### Gene expression assessment by RT-PCR

**Fig. 4:**
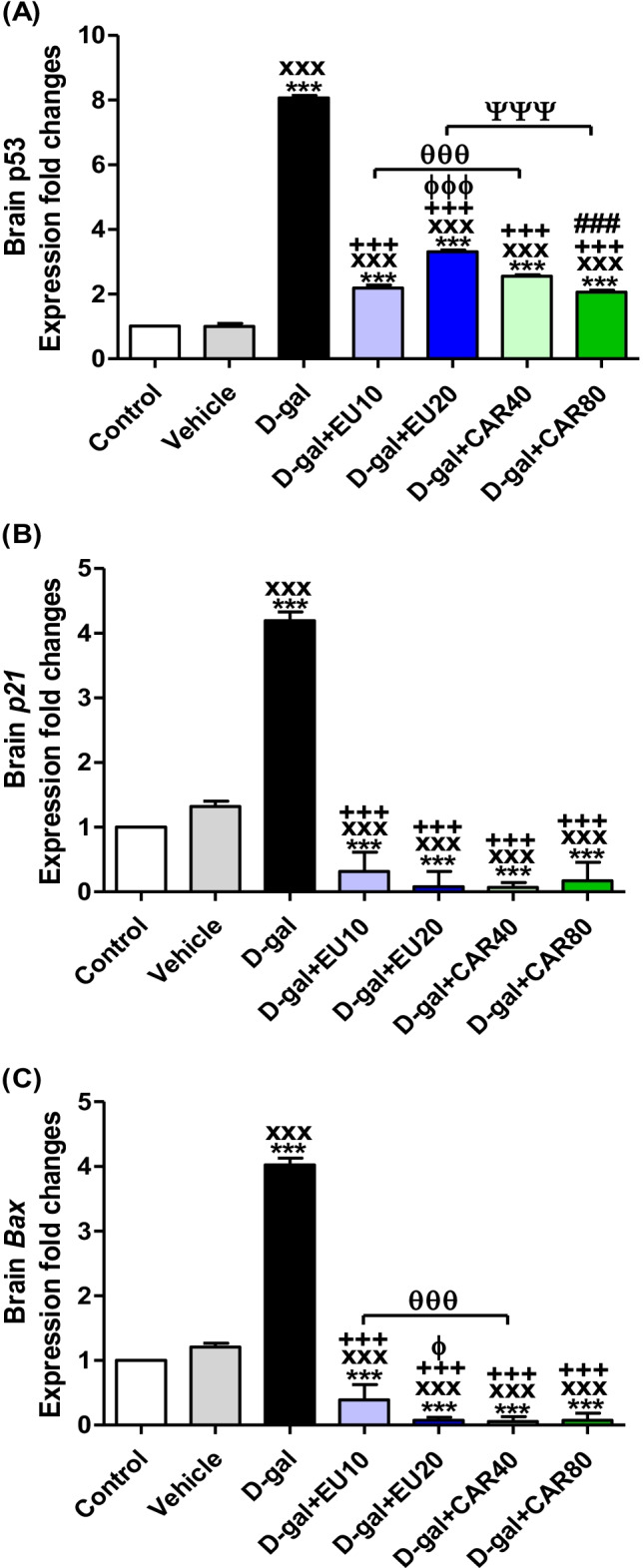
mRNA relative fold change expression of brain tissue. **A** *p53*, **B** *p21*, and **C** Bcl-2-associated X protein (*Bax*). Data were analyzed with a one-way ANOVA followed by Tukey’s multiple comparison test. ^***^P < 0.001 vs the control. ^xxx^*P* < 0.001 vs Vehicle. ^+++^*P* < 0.001 vs d-gal. ^ɸ^*P* < 0.05 and ^ɸɸɸ^*P* < 0.001 vs d-gal+EU10. ^###^*P* < 0.001 vs d-gal+CAR40. ^θθθ^*P* < 0.001 for d-gal+EU10 and d-gal+CAR40 comparison. ^ΨΨΨ^*P* < 0.001 for d-gal+EU20 and d-gal+CAR80 comparison. Error bars represent mean ± SD. *n*= 4.

The brain *p53* mRNA expressions in the d-gal, d-gal+EU10, d-gal+EU20, d-gal+CAR40, and d-gal+CAR80 groups were significantly increased (*P* < 0.001) compared with the control and vehicle groups (Fig. 4A). Compared with the d-gal group, in the d-gal+EU10, d-gal+EU20, d-gal+CAR40, and d-gal+CAR80 groups, *p53* expressions were significantly decreased (*P* < 0.001).

**Fig. 5:**
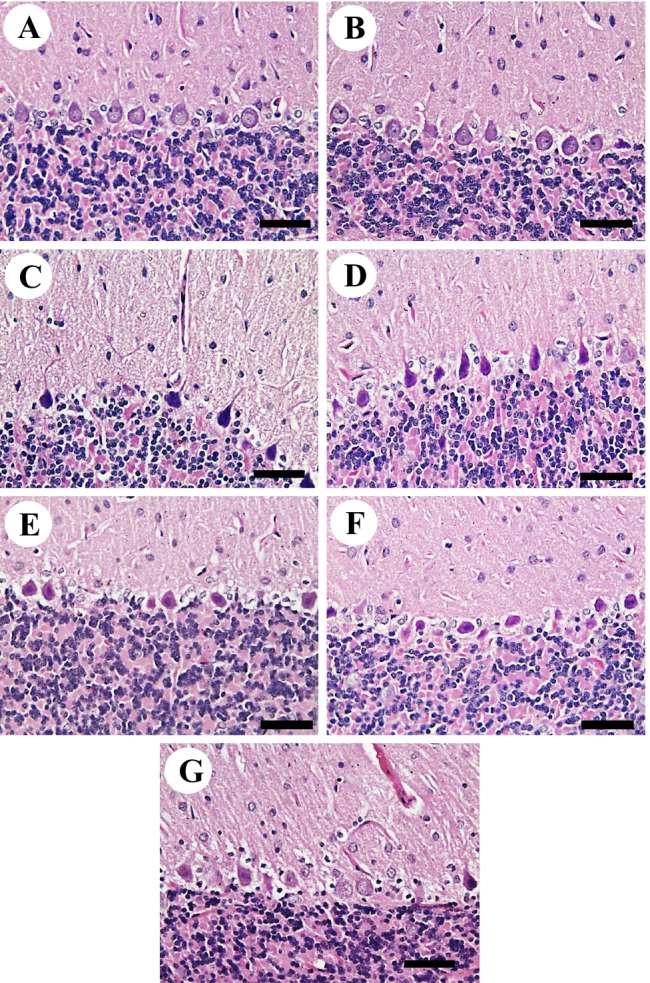
Histopathological examination of rats’ cerebellum. **A** Control. **B** Vehicle group. **C** d-gal group revealing necrosis of Purkinje cells in Purkinje cells layer. **D** d-gal+EU10. **E** d-gal+EU20. **F** d-gal+CAR40. **G** d-gal+CAR80. Scale bar= 50 μm.

On the other hand, its expression levels in d-gal+CAR40 were significantly raised (*P* < 0.001) compared with the d-gal+CAR80 group. In the d-gal+EU10 group, its level was a significant decrease (*P* < 0.001) in comparison with d-gal+EU20 and d-gal+CAR40 groups, while in the d-gal+EU20, its level showed a significant increase (*P* < 0.001) compared with the d-gal+CAR80 group (Fig. 5).

Brain *p21* mRNA expression levels in the d-gal group were significantly increased (*P* < 0.001) compared with the control group (Fig. 4B). In the d-gal+EU10, d-gal+EU20, d-gal+CAR40, and d-gal+CAR80 groups, the expression levels were significantly decreased (*P* < 0.001) compared with the d-gal group. In the d-gal group, its levels showed significant increases (*P* < 0.001) compared with the vehicle group, while its levels were significantly decreased (*P* <0.001) in d-gal+EU10, d-gal+EU20, d-gal+CAR40, and d-gal+CAR80 groups compared with the vehicle group.

The brain *Bax* mRNA expression in the d-gal group was significantly increased (*P* < 0.001) compared with the control group. On the other hand, in the d-gal+EU10, d-gal+EU20, d-gal+CAR40, and d-gal+CAR80 groups, its expression levels were significantly decreased (*P* < 0.001) in comparison with the control group. Also, the d-gal group was exhibited significant increases in *Bax* expression levels (*P* < 0.001) compared with the vehicle group (Fig. 4C). In the d-gal+EU10, d-gal+EU20, d-gal+CAR40, and d-gal+CAR80 groups, the brain *Bax* mRNA expression levels were significantly decreased (*P* < 0.001) in comparison with the d-gal and vehicle group. The d-gal+EU10 group showed a markedly raised (*P* < 0.05) expression level compared with the d-gal+EU20 group. Also, in the d-gal+EU10 group, its levels were significantly increased (*P* < 0.001) compared with the d-gal+CAR40 group (Fig. 4C).

### Histopathological assessment

Negative control and vehicle groups showed a normal cerebellar architecture that consisted of uniform molecular, granular and Purkinje cell layers (Fig. 5A, B). On the other hand, the d-gal group revealed loss and necrosis of Purkinje cells in the Purkinje cells and granular cells layers (Fig. 5C). d-gal+EU10 showed improvement in the number of Purkinje cells in the Purkinje cells layer with a lower number of pyknotic nuclei than the d-gal group (Fig. 5D). d-gal+EU20 and d-gal+CAR40 revealed a relatively normal cerebellar structure as a negative control group (Fig. 5D, F). d-gal+CAR80 showed improvement in the number of Purkinje cells in the Purkinje cells layer with a lower number of pyknotic nuclei than the d-gal group (Fig. 5G).

**Fig. 6:**
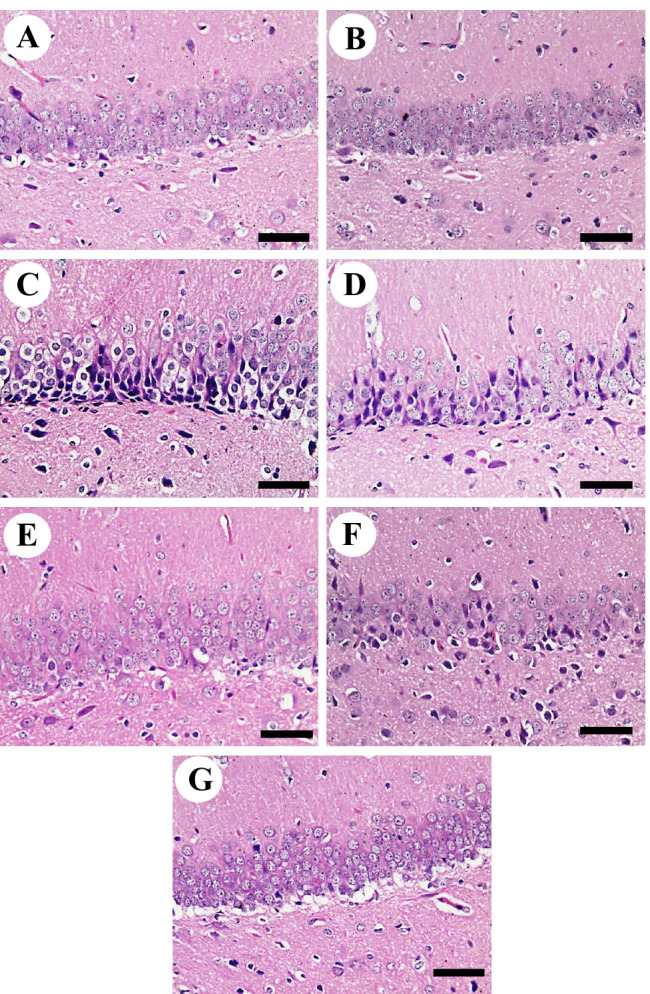
Histopathological examination of rats’ hippocampus. **A** control group. **B** vehicle group. **C** d-gal group revealing the necrosis of dentate gyrus neurons. Layers and numbers of hippocampal cells were decreased with enlarged intercellular space and disordered cells; especially, some cells exhibited shrink in volume, with pyknosis or rupture in nuclei. **D** d-gal+EU10. **E** d-gal+EU20. **F** d-gal+CAR40. **G** d-gal+CAR80. Scale bar= 50 μm.

Negative control and vehicle groups showed normal hippocampal architecture (Fig. 6A, B). On the other hand, the necrosis of dentate gyrus neurons was intensive in the d-gal group. Layers and numbers of hippocampal cells were decreased with enlarged intercellular space and disordered cells; especially, some cells exhibited shrink in volume, with pyknosis or rupture in nuclei (Fig. 6C). d-gal+EU10 showed improvement of hippocampal cells with lower necrosis than the d-gal group (Fig. 6D). d-gal+EU20 revealed a relatively normal hippocampal structure as the negative control group (Fig. 6E). d-gal+CAR40 revealed a relatively normal hippocampal structure as the negative control group (Fig. 6F). d-gal+CAR80 showed improvement of hippocampal cells with lower necrosis than the d-gal group (Fig. 6G).

## Discussion

The percentage of people aged 60 or older was reported to be 12.3% in 2015 and is predicted to increase to 21.5% of the world’s population by 2050, according to the UN World Population Prospects (Sander et al. 2015). Oxidative stress, a process characterized by the progressive loss of tissue/organ function, is the main inducer of aging (Shwe et al. 2018).

An exogenous dose of d-gal can induce aging effects in several organs by increasing oxidative stress, apoptosis, and inflammation (Rehman et al. 2017; El-Far et al. 2020b). The brain, due to high metabolism, high-fat content, and limited antioxidants protection mechanisms, is the organ most vulnerable to oxidant stress (Çakatay 2010).

**Fig. 7:**
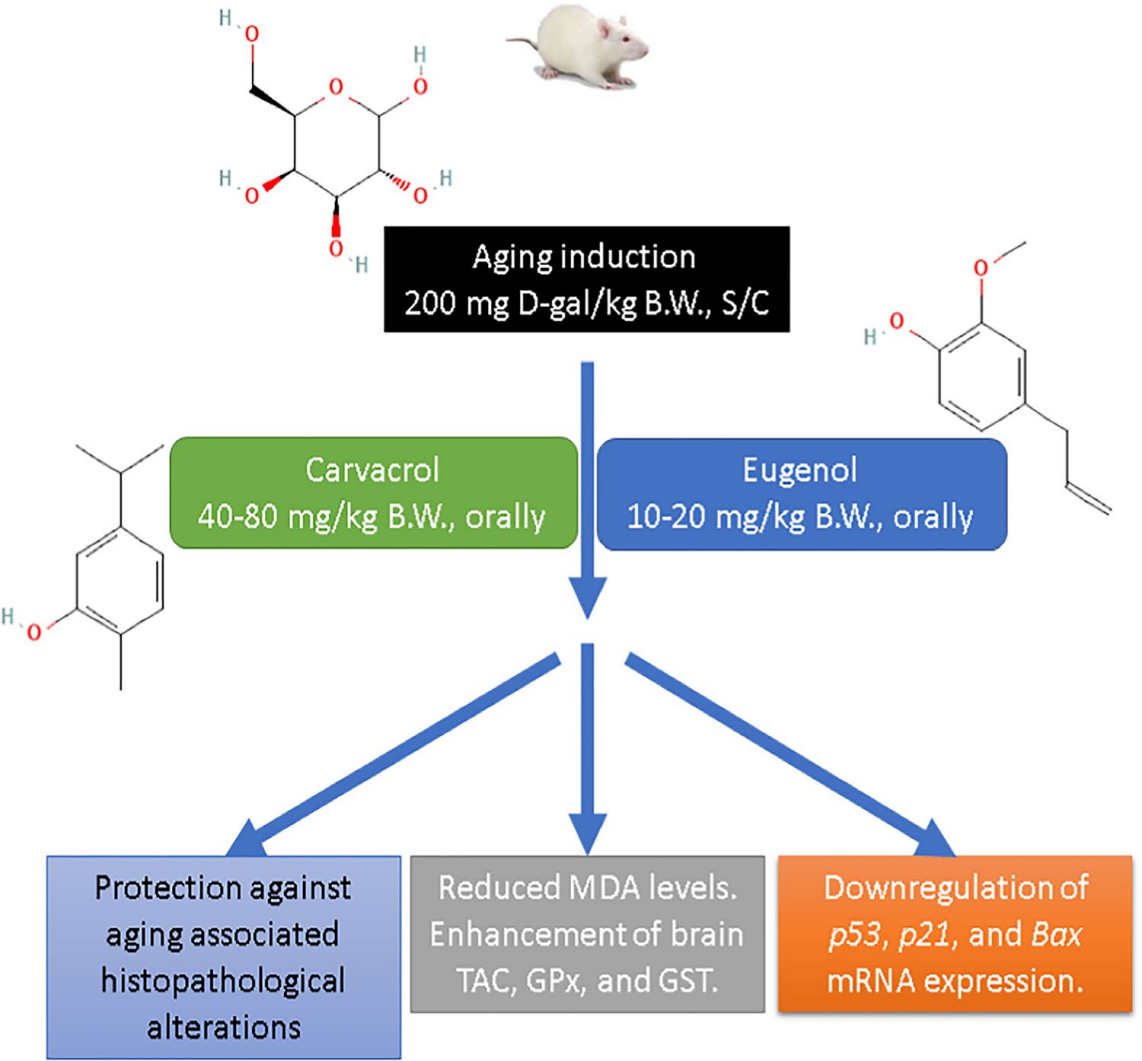
Eugenol (EU) and carvacrol (CAR) protective potential against oxidative stress induced by d-galactose (d-gal). Bax; Bcl-2-associated X protein. GPx; glutathione peroxidase. GST; glutathione S-transferase. MDA; malondialdehyde. TAC; total antioxidant capacity.

In the current study, EU and CAR were significantly attenuated the oxidative stress process in brain homogenates induced by d-gal. EU and CAR significantly increased the activities of GPx and GST, antioxidant enzymes as illustrated in Fig. 7. In the same context, EU induced neuroprotective potential against aluminum-induced oxidative stress by enhancing GPx activity (Mesole et al. 2020). In addition, combining acupuncture and eugenol enhanced learning-memory ability and antioxidation system of hippocampus in Alzheimer’s disease rats (Liu et al. 2013). Also, CAR protected against aluminum-induced oxidative stress (Baranauskaite et al. 2020) and Parkinson’s disease (Manouchehrabadi et al. 2020) through enhancement of antioxidant enzymes activities.

The d-gal causes a significant decrease in body weight when comparing the d-gal group with the control group. Numerous studies of experimentally induced aging reported that the d-gal decreased the body weight in rats (Chen et al. 2018) and mice (Suo et al. 2018). The current study showed that either EU or CAR administration to rats treated with d-gal could significantly restore body weight to near normal during the treatment period (42 days). This observation is supported by the study of Harb et al. (2019) that showed this improvement in body weight gain in rats by a dose of 10 mg EU per kg B.W. Mohammadi et al. (2014) stated that EU of the clove essential oil could stimulate the proliferation and growth of *Lactobacillus* that causes changes in villi of the small intestine and effectively improve broiler growth performance. Also, CAR in increasing the animal performance as in Wister rats (Rajan et al. 2015), broiler (Hashemipour et al. 2013), and juvenile rainbow trout (Ahmadifar et al. 2011).

According to the biochemical level, a notable reduction was observed in the serum cholesterol levels of treated groups with either EU or CAR. This could be due to their hypocholesterolemic activity of them. All doses of either EU or CAR cause a reduction in the serum total TAG levels when orally administered to the d-gal-treated group daily 42 days, although this decrease was not high. Elbahy et al. (2015) and Karam et al. (2015) confirmed our findings that EU and CAR reduced serum TAG and cholesterol levels.

As a response to the DNA damage, p53 becomes functionally active. It initiates either a reversible cell cycle arrest, cell death (apoptosis), or irreversible cell cycle arrest (cellular senescence), leading to aging (Rodier et al. 2007). p53 regulates a complex antiproliferative transcriptional program that is related to senescence, which induces the transcription of the cyclin-dependent kinase inhibitor (CDKi) p21 (Macip et al. 2002), which blocks CDK2 activity, resulting in cell cycle exit (Herranz and Gil 2018).

According to the molecular level, this study showed the effect of d-gal injection on the brain *p53* and *p21* mRNA expression levels. There was a significant elevation in the brain *p53* and *p21* mRNA expression in response to the d-gal injection. El-Far et al. (2020a) recognized significant upregulations of *p53* and *p21* expressions in d-gal treated rats. Also, Sun et al. (2018) declared an upregulation of *p21* upregulation in mice injected with d-gal. d-gal causes oxidative stress at high levels via the accumulation of ROS, stimulates free radical production, and reduces antioxidant enzyme activities (Xu et al. 2009). Oxidative damage and inflammation play critical roles in mediating the age-related alterations in different organs such as the brain, muscle, and kidney (Wei et al. 2005).

The current results showed significant decreases in the brain *p53* mRNA expression in EUR- and CAR-treated groups than the d-gal group, but its levels were still higher than the control groups. As for the brain, *p21* and *Bax* mRNA expression levels were significantly decreased in EUR- and CAR-treated groups than control and d-gal groups. d-gal induces senescence of glioblastoma cells with upregulation of *p53* (Xu et al. 2020). Liu et al. (2018) recognized the upregulation of *p53* and *p21* in the hippocampus of mice treated with d-gal. Also, d-gal induced significant elevations in *p53*, *p21*, and *Bax* in rats’ pancreas and kidneys (El-Far et al. 2020b).

Manikandan et al. (2010, 2011) reported that the administration of EU induced apoptosis via the mitochondrial pathway by modulating the Bcl-2 family proteins, while according to the CAR, Potočnjak and Domitrović (2016) stated its role in decreasing the expression of *p53* and *p21* expressions against cisplatin-induced toxicity in mice.

In the present study, *Bax* mRNA expression level exhibited a significant increase due to the d-gal. In many studies, Shahroudi et al. (2017) and Xu et al. (2016) reported a significant elevation in *Bax* expression in brain tissues of mice treated with d-gal, while the *Bax* mRNA expression level exhibited significant decreases in rats treated with EU and CAR. Júnior et al. (2016) demonstrated that the EU promotes *Bax* overexpression in cervical cancer cells. Shoorei et al. (2019) reported that CAR decreased the *Bax* expression in testicular tissue of adult diabetic rats. Also, Sadeghzadeh et al. (2018) mentioned that the *Bax* expression reduced by the effect of CAR in the hypertrophied heart in rats.

In the current study, d-gal induced necrosis of Purkinje cells in Purkinje cells layer of rats’ cerebellum and necrosis of dentate gyrus neurons hippocampus. In contrast, these necroses were defeated in EU- and CAR-supplemented groups. Chiroma et al. (2018) stated that d-gal and aluminum chloride-induced marked neuronal loss in rats’ hippocampus.

Regarding the protective effects of the EU and CAR against the histopathological changes in the cerebellum and hippocampus, the present study is considered the first recognized significant alleviation in the brain’s histopathological changes.

## Conclusion

The oxidative stress hypothesis remains possibly the basis of aging-associated cellular alterations. EU and CAR in a dose-dependent manner potentially attenuated the oxidative stress induced by d-gal in the brain tissues of rats through downregulation of aging markers (*p53* and *p21*) and apoptotic marker (*Bax*) with an enhancement of the antioxidant status of brain tissues. Our results suggest that the EU and CAR successfully alleviated the aging of rats’ brain tissues, rendering them promising natural anti-aging supplements.

## Data Availability

All data generated or analyzed during this study are included in this published article.
