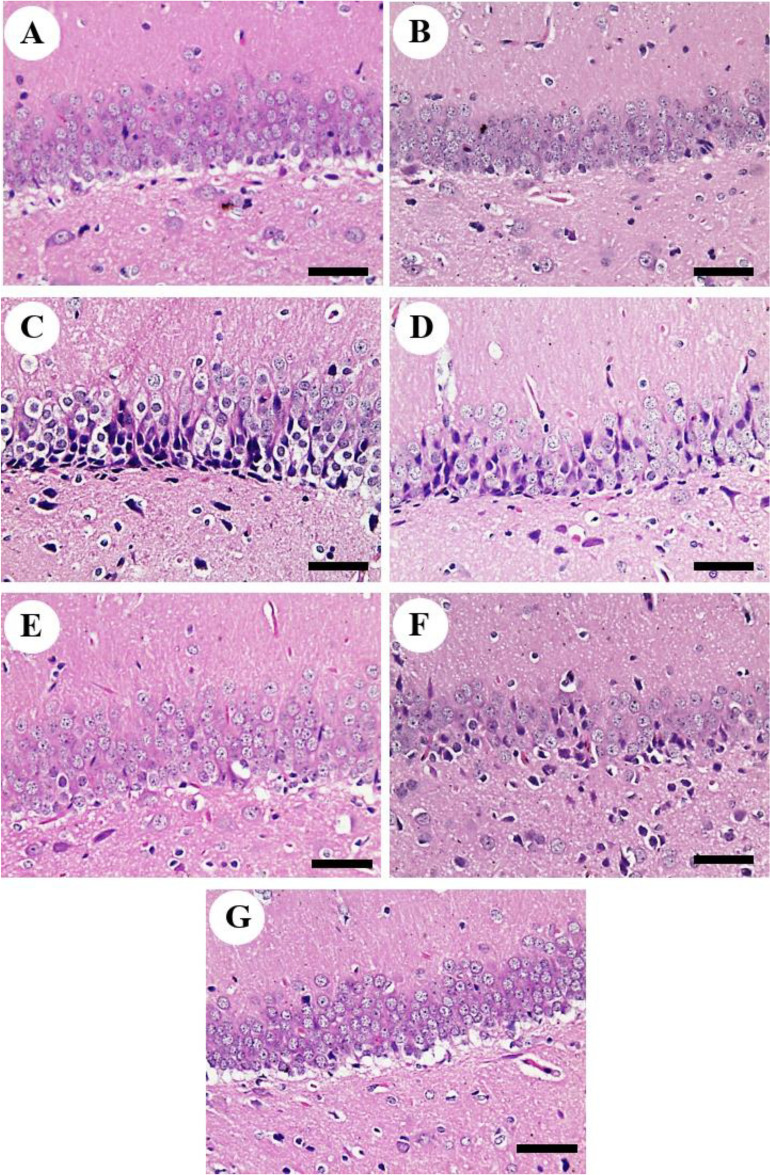# Correction to: Eugenol and carvacrol attenuate brain D-galactose-induced aging-related oxidative alterations in rats

**DOI:** 10.1007/s11356-024-34985-1

**Published:** 2024-09-17

**Authors:** Ali H. El‑Far, Hadeer H. Mohamed, Doaa A. Elsabagh, Shymaa A. Mohamed, Ahmed E. Noreldin, Soad K. Al Jaouni, Abdelwahab A. Alsenosy

**Affiliations:** 1 Department of Biochemistry, Faculty of Veterinary Medicine, Damanhour University, Damanhour, 22511 Egypt; 2 Molecular Biology, Molecular biology unit, Medical Technology Center, Medical Research Institute, Alexandria University, Alexandria, Egypt; 3 Histology and Cytology Department, Faculty of Veterinary Medicine, Damanhour University, Damanhour, 22511 Egypt; 4 Department of Hematology/Pediatric Oncology, Yousef Abdulatif Jameel Scientific Chair of Prophetic Medicine Application, Faculty of Medicine, King Abdulaziz University, 21589 Jeddah, Saudi Arabia

**Correction to: Environmental Science and Pollution Research (2022) 29:47436–47447**

10.1007/s11356-022-18984-8

The authors regret for Post-Publication Image Correction.

Unfortunately, we found a non-intended mis-upload to the image ‘‘A, control group’’ in Figure 6. We need to replace it with the correct Figure 6.

New Figure 6